# Supplementary material for: Anomalous excitonic phase diagram in band-gap-tuned Ta2Ni(Se,S)5
Source: Nat Commun. 2023 Nov 18;14:7512. doi: 10.1038/s41467-023-43365-1 (PMC10657405; doi:10.1038/s41467-023-43365-1)
Supplement: Supplementary file 1 — Supplementary Information [file 41467_2023_43365_MOESM1_ESM.pdf]

Supplementary Information

Anomalous excitonic phase diagram in band-gap-tuned $Ta_2Ni(Se,S)_5$

Cheng Chen,[1,2,*] Weichen Tang,[3,4,*] Xiang Chen,[3,4] Zhibo Kang,[2] Shuhan Ding,[5] Kirsty Scott,[2] Siqi Wang,[2] Zhenglu Li,[3,4,6] Jacob P.C. Ruff,[7] Makoto Hashimoto,[8] Dong-Hui Lu,[8] Chris Jozwiak,[9] Aaron Bostwick,[9] Eli Rotenberg,[9] Eduardo H. da Silva Neto,[10] Robert J. Birgeneau,[3,4,11] Yulin Chen,[1] Steven G. Louie,[3,4,11,†] Yao Wang,[5,12,‡] and Yu He[2,§]

[1]*Department of Physics, University of Oxford, Oxford, OX1 3PU, United Kingdom*

[2]*Department of Applied Physics, Yale University, New Haven, Connecticut 06511, USA*

[3]*Physics Department, University of California, Berkeley, California 94720, USA*

[4]*Materials Sciences Division, Lawrence Berkeley National Lab, Berkeley, California 94720, USA*

[5]*Department of Physics and Astronomy, Clemson University, Clemson, South Carolina 29631, USA*

[6]*Mork Family Department of Chemical Engineering and Materials Science, University of Southern California, Los Angeles, California 90089, USA*

[7]*Cornell High Energy Synchrotron Source, Cornell University, Ithaca, New York 14853, USA*

[8]*Stanford Synchrotron Radiation Lightsource, SLAC National Accelerator Laboratory, Menlo Park, California 94025, USA*

[9]*Advanced Light Source, Lawrence Berkeley National Laboratory, Berkeley, California 94720, USA*

[10]*Department of Physics, Yale University, New Haven, Connecticut 06511, USA*

[11]*Department of Materials Science and Engineering, University of California, Berkeley, California 94720, USA*

[12]*Department of Chemistry, Emory University, Atlanta, GA 30322, USA*

(Dated: October 25, 2023)

SUPPLEMENTARY NOTE 1: COMPLETE DATA OF RESISTIVITY, XRD, ARPES AND EDX MEASUREMENTS

Resistivity measurements were performed on each $Ta_2Ni(Se,S)_5$ sample with different S-doping levels, and the result is illustrated in Supplementary Fig. 1a. Similar temperature evolution is evidenced as the sample becomes more insulating towards low temperatures. However, the resistivity curve does not show clear signs of metal-to-insulator transition. The structural symmetry-breaking phase transition only manifests into a small kink, for instance around $T_s = 329$ K in the pristine Ta_2NiSe_5. This feature can be better visualized as the peak in activation energy Δ of the resistivity ρ, shown in Supplementary Fig. 1b. Such peaks are only evidenced in samples with S-doping levels below 72% (result plotted in Fig. 2b of the main text). Therefore, we note that transport measurement is not a direct measurement of the structural phase transition.

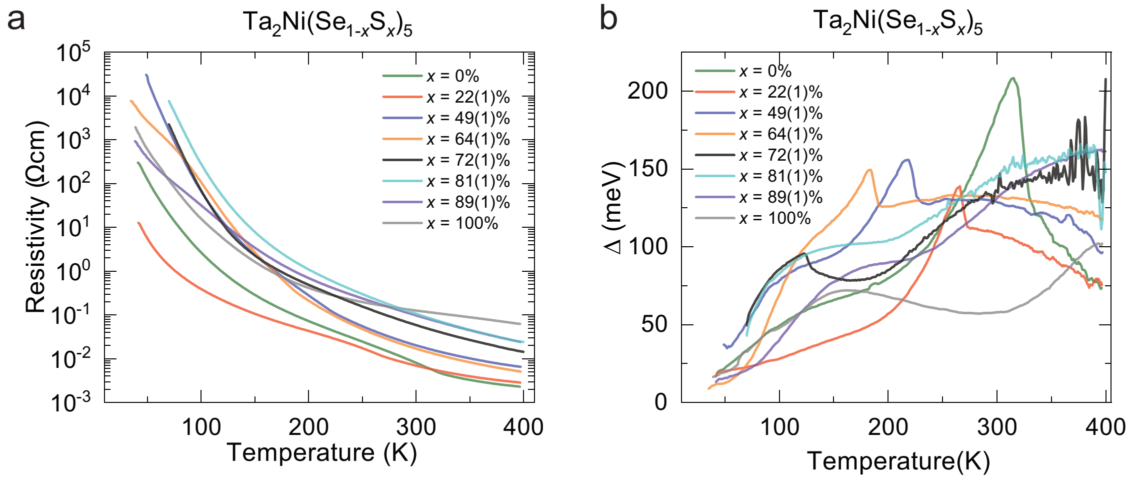

Supplementary Fig. 1. **Resistivity measurement of $Ta_2Ni(Se,S)_5$. a** Temperature-dependent resistivity of $Ta_2Ni(Se,S)_5$. **b** The temperature dependence of the activation energy Δ of the resistivity ρ in **a** given by $\Delta = -k_B T^2(\partial \ln \rho/\partial T)$. The actual S doping level x of each sample is determined by energy-dispersive x-ray spectroscopy, in which the typical variation of x is less than 1%.

Complete data for high-resolution synchrotron-based XRD measurement on $Ta_2Ni(Se,S)_5$ family is illustrated in Supplementary Fig. 2. Crossing the second-order structural phase transition, the system turns from the high-temperature orthorhombic phase to the low-

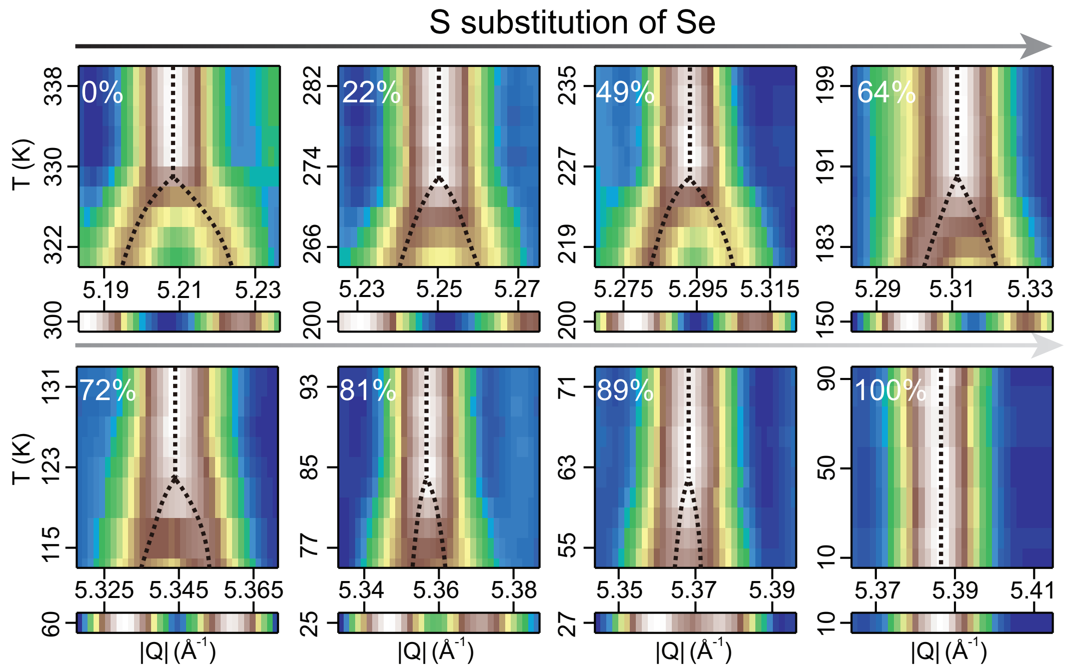

Supplementary Fig. 2. **High energy X-ray diffraction data of $Ta_2Ni(Se,S)_5$.** Evolution of the Bragg peak ± 2 4 8 as a function of temperature for samples with different S-doping levels. The splitting of the peak marks the structural phase transition from the high-temperature orthorhombic phase to the low-temperature monoclinic phase. No structural transition is observed in Ta_2NiS_5 within the resolution limit of the experiment (about 0.01 Å^{-1}). Black dashed lines are guides for the eyes of the Bragg peak positions.

temperature monoclinic phase, and some Bragg peaks, for instance the 2 4 8 Bragg peak shown here, split into two. Such splitting is evidenced in all samples with different S-doping levels, except for Ta_2NiS_5 , where no structural transition is observed within the resolution limit of the experiment. The temperature where the splitting starts marks the structural phase transition point deduced from XRD measurement and the result is summarized in Fig. 2a of the main text. On the other side, the evolution of β angle, i.e. structural order parameter in the low-temperature monoclinic phase, can be deduced from the separation of the split peak, and the result is plotted in Fig. 2b of the main text.

High statistics and energy resolution ARPES measurements are performed on $Ta_2Ni(Se,S)_5$ family at both the high-temperature orthorhombic phase and the low-temperature monoclinic phase (except for at 100% S-doping where no structural phase transition is observed) to trace the evolution of electronic structure with S-doping. Since the structural phase transi-

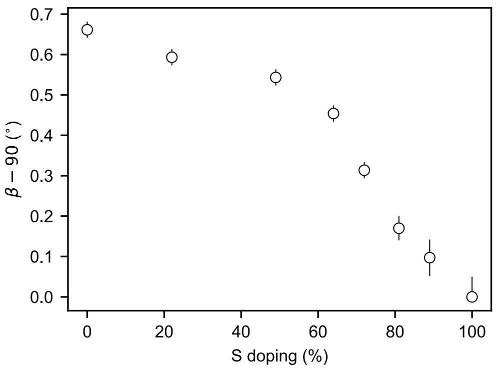

Supplementary Fig. 3. **Structural order parameter at the temperature of ARPES measurement** The amplitude of the deviation of the β angle from 90° at the temperature of ARPES measurement, as compared to the maximum value. No observable structural phase transition is observed at 100% S doped compound (Ta_2NiS_5).

tion is second order in nature, we plot the structural order parameter at the temperature of ARPES measurement in Supplementary Fig. 3, to ensure that visible features of the monoclinic phase have already developed. Linear horizontal (LH) and linear vertical (LV) incident beams from synchrotron radiation are employed, selectively probing bands with different orbital origins, which in this case highlight the conduction and valence bands respectively. The complete data is illustrated in Supplementary Fig. 4. For the high-temperature data, we divide the resolution-convolved Fermi-Dirac function to restore the spectra up to ∼150 meV above the Fermi level. For the low-temperature data, as the samples become insulating and the Fermi level is not pinned at a well-defined position, we aligned the spectra of different samples to the top of the valence band to ensure a direct comparison. For better visualization of the ARPES result, we combined the intensity of LH and LV channels with equal contributions in blue and red colors and plotted in Fig. 3 of the main text.

To check the spatial homogeneity of sulfur doping within each doped sample, we performed energy dispersive X-ray spectroscopy (EDX) mapping over the sample surface, and Supplementary Fig. 5 shows the results on the select samples with 64% and 72% S-doping levels. We find that the S-doping level within each sample is homogeneous with an average variation of up to ±2% between regions of 10-20 microns lateral dimensions, which matches our smallest ARPES beam spot sizes.

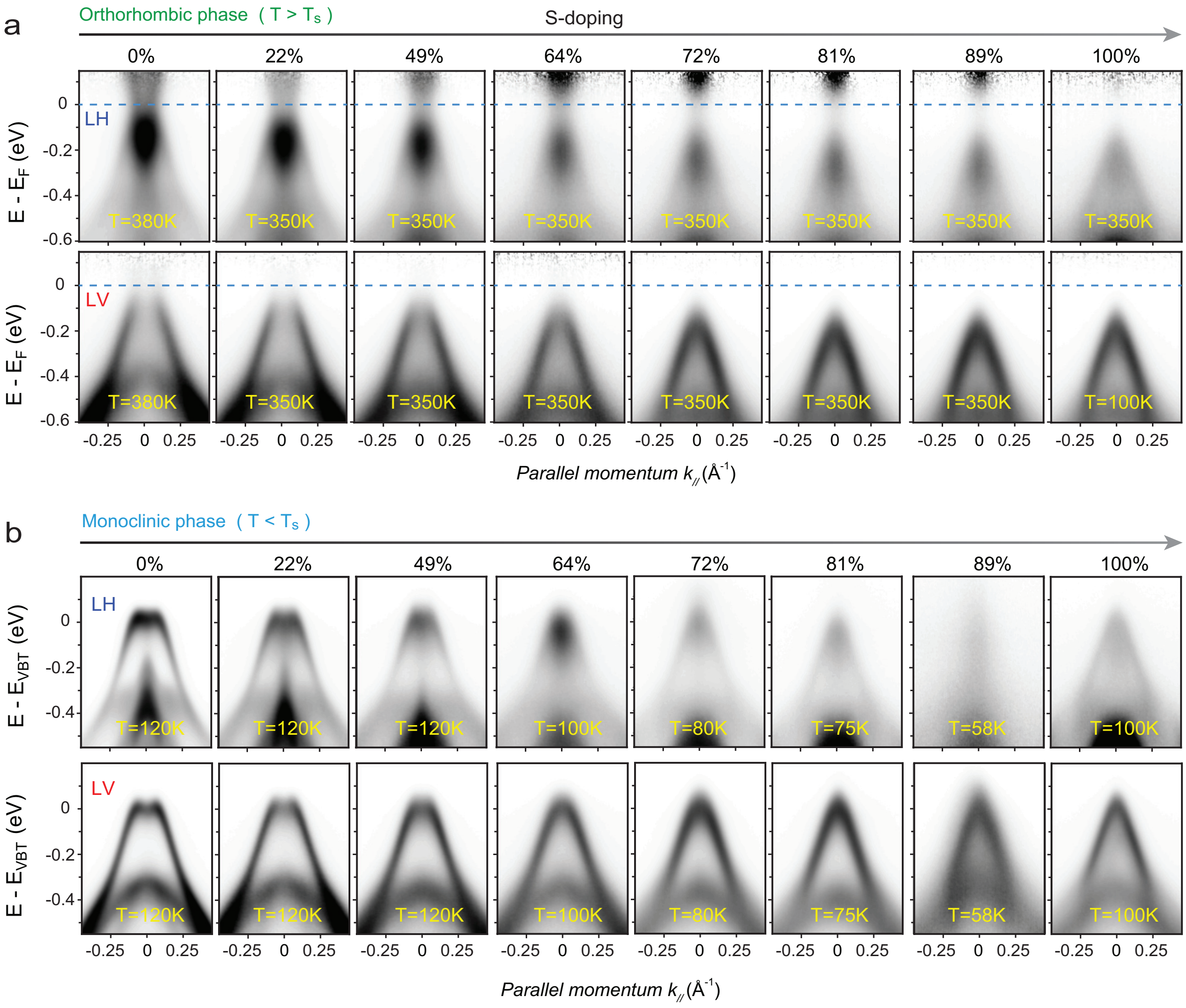

Supplementary Fig. 4. **Photoemission data of $Ta_2Ni(Se,S)_5$.** **a** Photoemission spectra along $X - \Gamma - X$ direction of $Ta_2Ni(Se,S)_5$ taken in high-temperature orthorhombic phase. LH (linear horizontal) and LV (linear vertical) denote the polarization of the incident photon beam. **b** Same as **a** but for spectra taken in low-temperature monoclinic phase, except for the 100% sulfur substituted compound (Ta_2NiS_5) where no sign of a structural phase transition is observed in the XRD measurement.

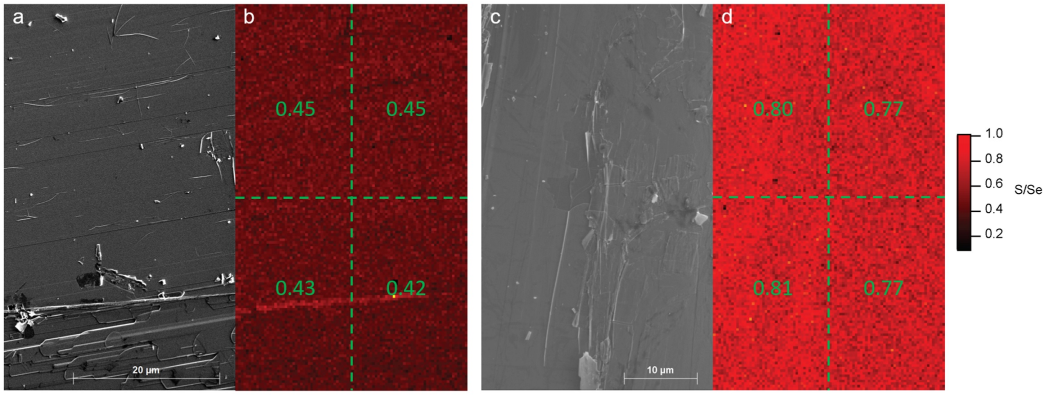

Supplementary Fig. 5. **Spatial homogeneity of S doping in $Ta_2Ni(Se,S)_5$. a(c)** SEM image of the surface of a sample with 64% and 72% S-doping. **b(d)** The false-color plot of the ratio between the intensity of sulfur K-alpha peak and selenium L peak corresponding to a(c). The green numbers on b(d) indicate the ratio between the intensity of sulfur K-alpha peak and selenium L peak averaged in the quarters segmented by the green dashed lines. The variation of the actual S-doping level determined from our EDX measurements in each sample is typically less than 1%.

SUPPLEMENTARY NOTE 2: FITTING OF PHOTOEMISSION SPECTRA

To estimate the band overlap/gap E_g in the high-temperature ARPES spectra, we fitted both the conduction and valence band dispersion. As illustrated in Supplementary Fig. 6, the spectra were first normalized along the energy direction to compensate for the spectra weight depletion around the Fermi level, resulting from the pseudogap state [1]. Then, the dispersion of the bands was traced by peak-fitting the energy distribution curves (EDCs) or momentum distribution curves (MDCs) of the ARPES spectra. The conduction band is fitted with the parabolic curve (blue dashed line), and the valence band is fitted using 3 different band shapes: linear (dashed yellow line), hyperbola (red line), and DFT result (dashed orange line). The upper and lower bound of E_g comes from the fitting result of the linear and DFT band shapes, respectively. In the case of quantifying low-temperature band back-bending momentum k_F, similarly, we trace the dispersion of the valence band by peak-fitting the EDCs and MDCs of the ARPES spectra. The $2k_F$ value is taken from the distance between the valence band top and the error comes from the momentum resolution of the ARPES measurement.

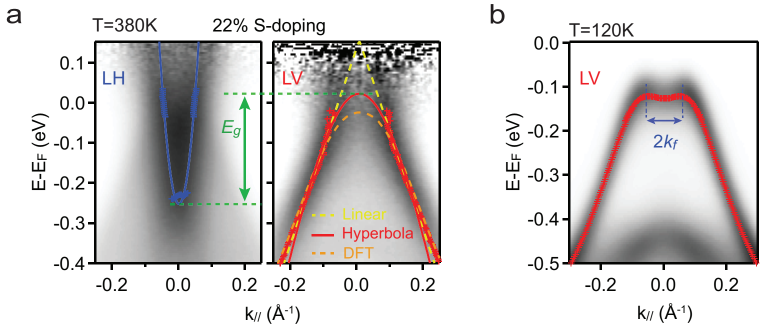

Supplementary Fig. 6. **Photoemission spectra fitting.** **a** High-temperature photoemission spectra of a 22% S-doped sample in both LH (linear horizontal) and LV (linear vertical) channels, highlighting conduction and valence band respectively. The spectra are normalized along the energy axis to remove the effect of the pseudogap state [1]. The "+" marks are deduced from the peak fitting of energy distribution curves (EDCs) or momentum distribution curves (MDCs), which were then fitted by hyperbola to extract the size of the band overlap E_g. The upper (lower) bound of E_g is estimated from the fitting of the valence band with a linear (normalized DFT dispersion) band shape. **b** Low-temperature photoemission spectra of the 22% S-doped sample in LV channel. Band dispersion is deduced from the peak fitting of EDCs or MDCs, from which the size of $2k_F$ is extracted.

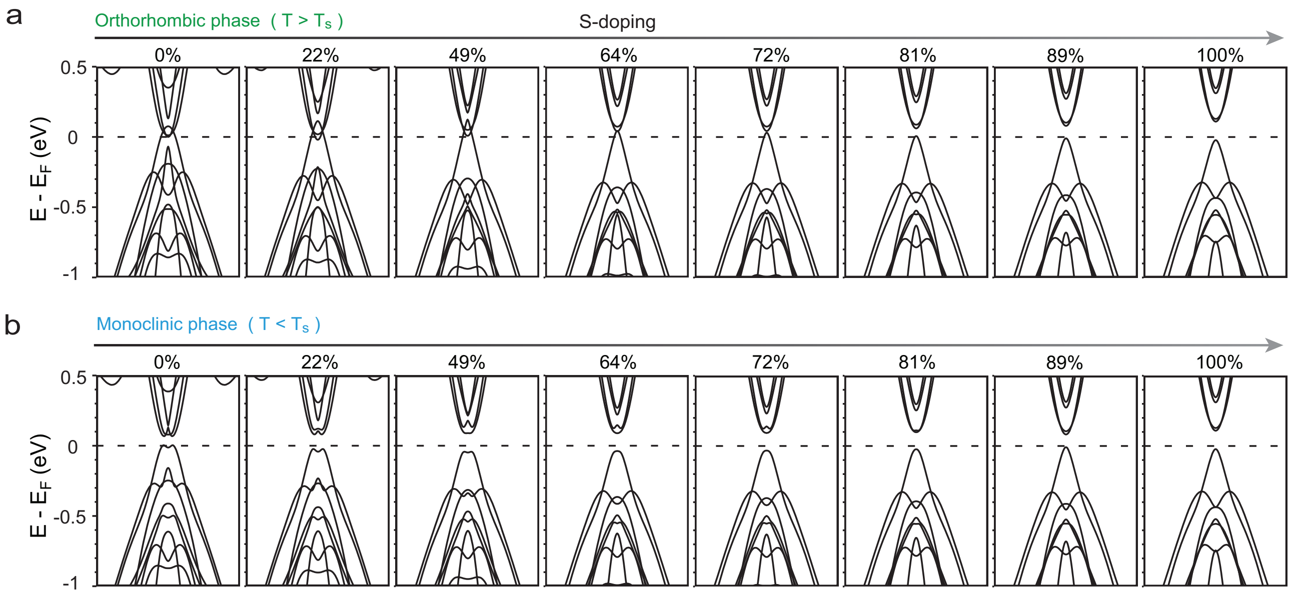

Supplementary Fig. 7. **DFT calculation of $Ta_2Ni(Se,S)_5$.** Calculated band structure along $X - \Gamma - X$ direction of $Ta_2Ni(Se,S)_5$ in **a** orthorhombic lattice structure, **b** monoclinic lattice structure, respectively.

SUPPLEMENTARY NOTE 3: DFT CALCULATION

Supplementary Fig. 7 shows complete data of DFT calculation on $Ta_2Ni(Se,S)_5$ in both the orthorhombic and the monoclinic phases. The orthorhombic phase is achieved by imposing the system's symmetry to conform to the orthorhombic structure, while the monoclinic phase is attained through full relaxation of the structure. The result is overlayed on the ARPES spectra in Fig. 3a of the main text. The band overlap/gap E_g in the orthorhombic phase and the band back back-bending momentum k_F in the monoclinic phase are also deduced and the result is plotted in Fig. 3c-d of the main text.

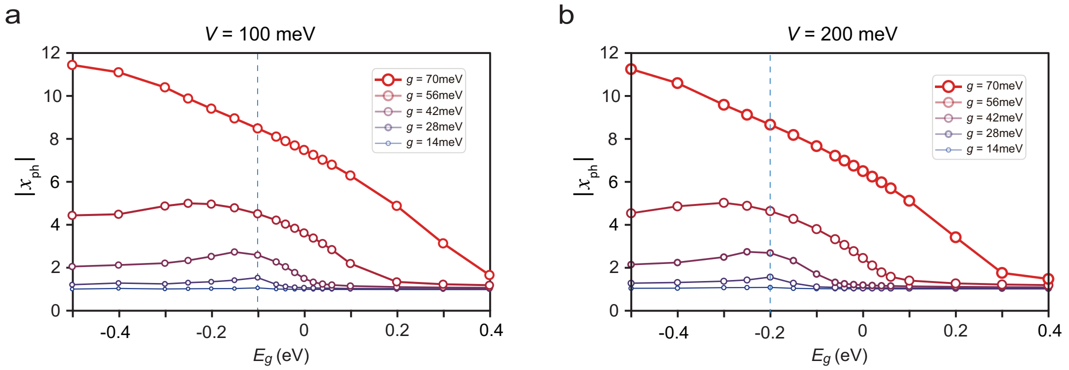

Supplementary Fig. 8. **Influence of direct Coulomb interaction V.** The calculated mean-average lattice displacement $|x_{\mathrm{ph}}|$ as a function of the band gap E_g at various electron-phonon coupling strengths, with finite Coulomb interaction (a) $V = 100\,\mathrm{meV}$. and (b) $V = 200\,\mathrm{meV}$. (The $V = 0$ case is shown in Fig. 4 of main text).

SUPPLEMENTARY NOTE 4: IMPACT OF THE COULOMB INTERACTION

To understand the impact of Coulomb interactions [the V in Eq. (1) of the main text], we incorporate non-zero interaction terms into the simulation of the many-body system. Supplementary Fig. 8 shows the mean-average lattice displacement $|x_{\mathrm{ph}}|$ evaluated using ED simulations for systems with $V = 100\,\mathrm{meV}$ and $200\,\mathrm{meV}$. While considering the upper limit of the experimentally determined Coulomb interaction around $70\,\mathrm{meV}$ [1], we allow for adjustments within a certain range to accommodate potential variations induced by S-doping. Compared to the Fig. 4 of the main text, the dependence of lattice displacement on E_g presented in Supplementary Fig. 8 shows similar behavior with an overall shift equal to the Coulomb interaction V. Such a shift can be better characterized by the hump positions in the weak-coupling limit. The overall move of the critical E_g reflects a relative motion between two bands caused by the Coulomb interaction V and this phenomenon can be attributed to the Hartree component.

In order to gain deeper insights into the influence of the parameter V, we perform a mean-field (MF) analysis on the many-body Hamiltonian [Eq. (1) of the main text]. Specifically, we consider the MF excitonic order parameter $\Delta_{\mathrm{ext}} = \sum_{i\sigma}\langle f^\dagger_{i\sigma} c_{i\sigma}\rangle/2N$ and $\Delta'_{\mathrm{ext}} = \sum_{i\sigma}\langle f^\dagger_{i\sigma} c_{i+1,\sigma}\rangle/2N$, which are identical when the reflection symmetry is preserved, and the MF lattice order parameter $X_{\mathrm{ph}} = \langle x_{\mathrm{ph}}\rangle$. The MF decomposition of Eq. (1) is as

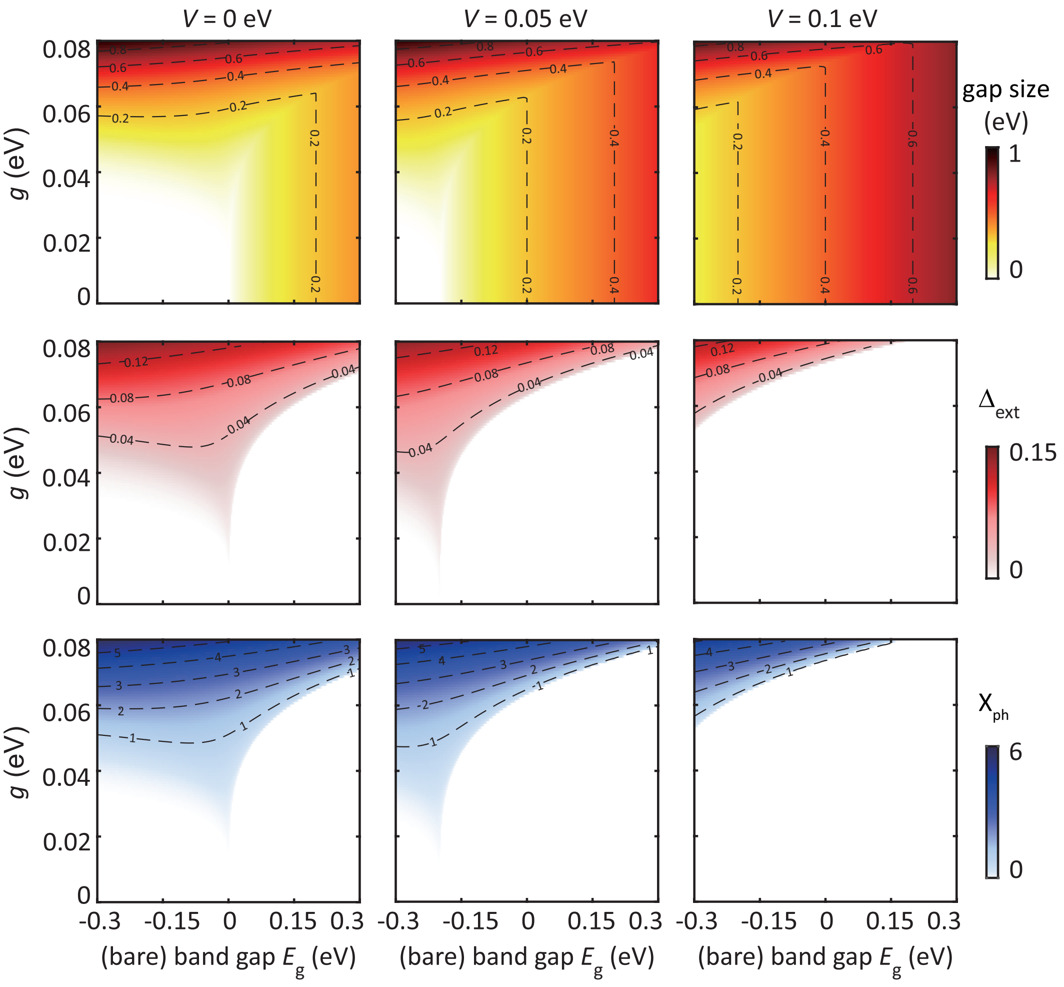

Supplementary Fig. 9. **Mean-field solutions for various interactions and bare band gap sizes.** Upper panels: Direct mean-field gap as a function of electron-phonon coupling g and bare band gap E_g, for interacting electrons with $V = 0$ (left), $V = 0.05\,\text{eV}$ (middle), and $V = 0.1\,\text{eV}$ (right). Middle and lower panels: Mean-field order parameter for electrons Δ_{ext} (red) and lattice X_{ph} (blue).

follows

$$\mathcal{H}_{\text{MF}} = \sum_{k\sigma}(c^\dagger_{k\sigma},\, f^\dagger_{k\sigma}) \begin{pmatrix} \varepsilon^c_k + 2V\sum_{i\sigma'}\langle n^f_{i\sigma'}\rangle/N & gX_{\text{ph}} - V(\Delta_{\text{ext}} + \text{e}^{-\text{i}k}\Delta'_{\text{ext}}) \\ gX_{\text{ph}} - V(\Delta_{\text{ext}} + \text{e}^{-\text{i}k}\Delta'_{\text{ext}}) & \varepsilon^v_k + 2V\sum_{i\sigma'}\langle n^c_{i\sigma'}\rangle/N \end{pmatrix} \begin{pmatrix} c_{k\sigma} \\ f_{k\sigma} \end{pmatrix}$$
$$- \sum_{i\sigma\sigma'}\langle n^c_{i\sigma}\rangle\langle n^f_{i\sigma}\rangle + V(\Delta^2_{\text{ext}} + \Delta'^{\,2}_{\text{ext}}) + \frac{1}{2}N\omega_0 X^2_{\text{ph}}, \tag{1}$$

where the V contributions to the diagonal terms reflect the Hartree shifts between two bands, and X_{ph} is calculated by minimizing the ground-state energy.

Solving the mean-field Hamiltonian yields the single-particle gap, which is jointly determined by the Hartree shift and the hybridization arising from the (off-diagonal) order parameters. As shown in the upper panels of Supplementary Fig. 9, the single-particle gap depends on both the e-ph coupling g and the bard band gap E_g. While a positive E_g, reflecting the S-doping in $Ta_2Ni(Se,S)_5$, enhances the gap, this resultant gap opening is distinct from the hybridization gap induced by symmetry breaking. This distinction can be reflected in the lower panels, where the order parameters Δ_{ext} and X_{ph} are strongly suppressed by positive E_g. Due to this suppression, these order parameters display a dome-like structure centered around $E_g = 0$ in the case of a weak-coupling system. When the e-ph coupling is strong, however, the hybridization gap is beyond the influence of E_g, resulting in a monotonic reduction of order parameters as E_g increases. This behavior is consistent with the ED simulation depicted in the Fig. 4 of the main text.

When incorporating the Coulomb interaction V into this MF analysis, its contribution to the order parameters is found to be minor, reflected by the slight lowering of iso-intensity curves when contrasting the middle and left panels. Instead, its primary contribution lies in uniformly shifting the complete phase diagram towards smaller E_g values. Therefore, the $Ta_2Ni(Se,S)_5$ transition is primarily dictated by the strong e-ph coupling, as opposed to the electronic Coulomb interactions.

The inefficiency of the Coulomb interaction in forming excitons results from the small Fermi momentum and the opposite parity of the low-energy bands, which has been discovered by earlier experimental and theoretical studies [2, 3]. The mismatch of parity completely excludes the purely Coulomb-driven excitonic orders at $k = 0$. This explains the diminishment of order parameters for E_g, reflecting the reality close to the Ta_2NiS_5 side of the material. While such an exclusion is no longer exact for finite Fermi momenta, the small k_F in Ta_2NiSe_5 leads to limited excitonic instability, compared to the substantial Hartree shift caused by the Coulomb interaction [1]. In contrast, the intraband B_{2g} phonon connects the two bands directly and breaks the mirror symmetry. Thus, the coupling to lattice distortion exhibits more efficiency in forming excitons.

---

* These authors contributed equally to this work

† sglouie@berkeley.edu

‡ yao.wang@emory.edu

§ yu.he@yale.edu

[1] C. Chen, X. Chen, W. Tang, Z. Li, S. Wang, S. Ding, Z. Kang, C. Jozwiak, A. Bostwick, E. Rotenberg, M. Hashimoto, *et al.*, *Role of electron-phonon coupling in excitonic insulator candidate* Ta_2NiSe_5, arXiv preprint arXiv:2203.06817v2 (2022).

[2] G. Mazza, M. Rösner, L. Windgätter, S. Latini, H. Hübener, A. J. Millis, A. Rubio, and A. Georges, *Nature of symmetry breaking at the excitonic insulator transition:* Ta_2NiSe_5, Phys. Rev. Lett. **124**, 197601 (2020).

[3] M. D. Watson, I. Marković, E. A. Morales, P. Le Fèvre, M. Merz, A. A. Haghighirad, and P. D. King, *Band hybridization at the semimetal-semiconductor transition of* Ta_2NiSe_5 *enabled by mirror-symmetry breaking*, Phys. Rev. Res. **2**, 013236 (2020).
